# Inhibition of radiation-induced apoptosis by dexamethasone in cervical carcinoma cell lines depends upon increased HPV E6/E7

**DOI:** 10.1054/bjoc.2000.1114

**Published:** 2000-04-27

**Authors:** M C Kamradt, N Mohideen, E Krueger, S Walter, A T M Vaughan

**Affiliations:** Departments of Cell Biology, Neurobiology and Anatomy Radiotherapy, Loyola University Medical Center, Maywood, IL, 60153, USA

**Keywords:** HPV, dexamethasone, apoptosis, cervical carcinoma, p53

## Abstract

Through a glucocorticoid-responsive promoter, glucocorticoids can regulate the transcription of the human papillomavirus (HPV) E6 and E7 viral genes which target the tumour suppressor proteins p53 and Rb respectively. In C4-1 cells, the glucocorticoid dexamethasone up-regulated HPV E6/E7 mRNA and decreased radiation-induced apoptosis. In contrast, dexamethasone had no effect on apoptosis of cells that either lack the HPV genome (C33-a) or in which HPV E6/E7 transcription is repressed by dexamethasone (SW756). Irradiated C4-1 cells showed increased p53 expression, while dexamethasone treatment prior to irradiation decreased p53 protein expression. In addition, p21 mRNA was regulated by irradiation and dexamethasone in accordance with the observed changes in p53. Overall, glucocorticoids decreased radiation-induced apoptosis in cervical carcinoma cells which exhibit increased HPV E6/E7 transcription and decreased p53 expression. Therefore, in HPV-infected cervical epithelial cells, p53-dependent apoptosis appears to depend upon the levels of HPV E6/E7 mRNA. © 2000 Cancer Research Campaign


*British Journal of Cancer* (2000) **82**(10), 1709–1716
© 2000 Cancer Research Campaign
DOI: 10.1054/ bjoc.2000.1114, available online at http://www.idealibrary.com on IDEAL®

### Inhibition of radiation-induced apoptosis by dexamethasone in cervical carcinoma cell lines depends upon increased HPV E6/E7

**MC Kamradt[1,2], N Mohideen[2], E Krueger[2], S Walter[2] and ATM Vaughan[2]**

Departments of [1]Cell Biology, Neurobiology and Anatomy and [2]Radiotherapy, Loyola University Medical Center, Maywood, IL 60153, USA

**Summary** Through a glucocorticoid-responsive promoter, glucocorticoids can regulate the transcription of the human papillomavirus (HPV) E6 and E7 viral genes which target the tumour suppressor proteins p53 and Rb respectively. In C4-1 cells, the glucocorticoid dexamethasone up-regulated HPV E6/E7 mRNA and decreased radiation-induced apoptosis. In contrast, dexamethasone had no effect on apoptosis of cells that either lack the HPV genome (C33-a) or in which HPV E6/E7 transcription is repressed by dexamethasone (SW756). Irradiated C4-1 cells showed increased p53 expression, while dexamethasone treatment prior to irradiation decreased p53 protein expression. In addition, p21 mRNA was regulated by irradiation and dexamethasone in accordance with the observed changes in p53. Overall, glucocorticoids decreased radiation-induced apoptosis in cervical carcinoma cells which exhibit increased HPV E6/E7 transcription and decreased p53 expression. Therefore, in HPV-infected cervical epithelial cells, p53-dependent apoptosis appears to depend upon the levels of HPV E6/E7 mRNA. © 2000 Cancer Research Campaign

**Keywords**: HPV; dexamethasone; apoptosis; cervical carcinoma; p53

Over 95% of all cases of cervical carcinoma have been associated with the human papillomavirus (HPV), especially high risk types 16 and 18 (for review, see Lazo, 1999; Walbloomers et al, 1999). Papillomaviruses possess a double-stranded, circular DNA genome which contains viral early (E1–E7) and late (L1–L2) genes which are separated by a transcriptional control region, the upstream regulatory region (URR) (reviewed in Hoppe-Seyler and Butz, 1994). The HPV URR is directly upstream of the E6 and E7 genes, both of which have been shown to possess transforming growth potential in vitro, most likely by direct interference with p53 and retinoblastoma (Rb) (Werness et al, 1990). The tumour suppressor proteins p53 and Rb are both regulators of cell cycle progression. p53 mediates a G1/S checkpoint through transactivation of the p21 (WAF1/CIP1) gene, a cyclin-dependent kinase inhibitor (el-Deiry et al, 1993; Harper et al, 1993), while Rb sequesters E2F, a transcription factor responsible for transcription of genes essential for cell cycle progression (reviewed in Almasan et al, 1995). HPV E7 binds to the transcription factor binding pocket in Rb, thereby displacing E2F which promotes cell cycle progression through transactivation of target genes (Dyson et al, 1989). High-risk HPV E6 can abolish p53 function by sequestering p53 which inhibits its ability to bind DNA (Werness et al, 1990; Lechner and Laimins, 1994; Thomas et al, 1995) or E6 can target p53 for ubiquitin-dependent proteolysis (Scheffner et al, 1990; Band et al, 1991; Crook et al, 1991; Demers, et al, 1994). However, it has been shown that the presence of E6 in cervical cancer cell lines is not functionally equivalent to a loss of p53 by mutation or deletion. In contrast, despite the presence of E6 in HPV-infected cervical carcinoma cells, p53 is inducible and can activate the p53 target genes p21 and GADD45 in response to DNA damaging agents (Butz et al, 1995, 1996, 1999).

The URR of the HPV genome contains an enhancer region which is controlled by glucocorticoid response elements (GREs) (Gloss et al, 1991). Glucocorticoids, specifically dexamethasone, have been shown to activate the GRE of the HPV genome which increases transcription of E6/E7 in most cervical carcinoma cells, resulting in enhanced cell proliferation (Von Knebel Doeberitz et al, 1988). This increase in transcription has been shown in C4-1 cells where dexamethasone increases cell proliferation and tumorigenicity in nude mice, even though this does not occur in all cervical cancer cell lines, such as HeLa (Von Knebel Doeberitz et al, 1991, 1992). These effects have been interpreted as a consequence of the perturbation in p53 and Rb function when bound to E6 and E7 proteins. In contrast, transcription of HPV E6/E7 is blocked by dexamethasone in the SW756 cell line resulting in increased p53 levels and loss of the neoplastic phenotype of these cells (Von Knebel Doeberitz et al, 1994).

While dexamethasone has been shown to modulate cellular proliferation and tumorigenicity through increased transcription of E6/E7 and subsequent effects on p53 and Rb, the effect of glucocorticoids on apoptosis in cervical carcinoma cell lines has not been determined. However, studies have shown that E6 or E6/E7 can abrogate apoptosis in various cell systems (Pan and Griep, 1994; Puthenveettil et al, 1996; Thomas et al, 1996). Therefore, it was of interest to determine the effect of dexamethasone on apoptosis in HPV-transformed cells in comparison to cells in which E6/E7 are artificially overexpressed through transgenic or vector-mediated genetic manipulation. Since p53 plays an important role in the induction of apoptosis, it is possible that in HPV-positive cervical cancer cells, dexamethasone treatment increases E6/E7, thus compromising p53 function and leading to decreased

*Received 2 June 1999*
*Revised 5 November 1999*
*Accepted 23 January 2000*

*Correspondence to:* MC Kamradt, Hines Building, Room B303, Hines VA Hospital, Hines, IL 60141, USA

apoptosis following DNA damage. We show here that in the C4-1 cell line, dexamethasone decreased radiation-induced apoptosis, an effect that correlates with increased transcription of E6/E7 and subsequent reduction in levels of p53 protein and p21 mRNA. In contrast, dexamethasone did not alter radiation-induced apoptosis in cells in which HPV E6/E7 was repressed (SW756) or in cells which lack the HPV genome (C33-a). Overall, glucocorticoids decreased radiation-induced apoptosis only in cervical carcinoma cells which displayed increased transcription of HPV E6/E7 genes and decreased expression of p53.

### MATERIALS AND METHODS

### Cell culture

The human cervical carcinoma cell lines C4-1 and SW756 were cultured in Dulbecco's modified Eagle medium (DMEM; Sigma, St Louis, MO, USA) supplemented with 4.5 g $l^{-1}$ glucose and the C33-a cells were grown in Eagle minimum essential medium (EMEM; Sigma, St Louis, MO, USA). All media were supplemented with 4 mM L-glutamine, 100 U $ml^{-1}$ penicillin–streptomycin, and 10% fetal calf serum (FCS; Biologos, Naperville, IL, USA). All cultures were maintained in a 5% carbon dioxide ($CO_2$) atmosphere at 37°C. Cells were irradiated at 6 Gy using a dual head Cs-137 Gammacell 40 irradiator (Nordion International, Ontario, Canada). Dexamethasone (Decadron Phosphate, Merck, Sharp and Dohme, West Point, PA, USA) was added to cultures at a final concentration of 1 μM 30 min prior to irradiation.

### Northern blot analysis

Total RNA was isolated by the TriZol method (Gibco-BRL). Cells are scraped into the TriZol reagent which dissociates the nucleoprotein complex. Samples were chloroform extracted and the aqueous phase containing RNA was collected. RNA was precipitated with isoamyl alcohol and pelleted. The RNA pellet was washed with 75% ethanol, vacuum dried and dissolved in 100 μl RNAase free, DEPC-treated water. RNA was quantitated by spectrophotometry and 10 μg was run on an agarose gel containing formaldehyde. RNA was transferred to a nylon membrane (Bio-Rad) which was then probed with a $^{32}P$ radiolabelled cDNA probe (see below) prepared using the Prime-a-Gene Kit (Promega) according to the manufacturer's instructions. The washed membrane was exposed to X-ray film and developed using an autoprocessor.

### Preparation of the HPV 18 E6/E7 cDNA probe by PCR

In order to amplify the HPV-18 E6/E7 open reading frame, primers utilized were generously donated by Dr Lutz Gissman. The composition of these PCR primers was: E6/E7: 5′-ATGGCGCCCTTTGAGGATCC = nt 105–134; TTACT-GCTGGGATGCACACC-3′ = nt 907–888.

The E6/E7 probe was synthesized by polymerase chain reaction (PCR) from a plasmid containing the entire HPV 18 genome which was cloned into BlueScript at the *Hin*DIII site. A 50 μl PCR mix containing 20 mM Tris–HCl (pH 8.4), 50 mM potassium chloride (KCl), 1.5 mM magnesium chloride ($MgCl_2$), 0.2 mM E6-E7 primers, 2 U *Taq* DNA polymerase and 2 μl cDNA from the first strand synthesis reaction was made. Cycling was performed in a Perkin-Elmer DNA Thermal Cycler 480 as follows: 94°C 1 min, 64°C 1.5 min, 72°C 3 min for 25 cycles. To ensure that the probe was the appropriate size, PCR products were resolved by gel electrophoresis on a 1% agarose gel and were visualized by ethidium bromide staining.

### Preparation of the p21 cDNA probe

The p21 cDNA probe was the generous gift of Dr Manuel Diaz. The probe was labelled as described above.

### Apoptotic determination

*Morphology*

Cells were treated with 1 μM dexamethasone, irradiated with 6 Gy or treated with 1 μM dexamethasone for 30 min prior to irradiation. Dexamethasone remained in the culture medium throughout the experiment. Cells were treated on day 0 and collected to determine the per cent apoptosis on days 1, 2 and 4. Cells were trypsinized, washed in phosphate-buffered saline (PBS) and fixed in 100% methanol. For morphological analysis, cells were stained with propidium iodide (PI) and examined under the fluorescence light microscope. Photographs were taken and at least 200 cells were analysed for apoptotic morphology without knowledge of treatment. Apoptotic cells are those with fragmented or condensed nuclei, while live cells exhibit a round, intact nucleus.

*Annexin staining*

Annexin V binds to phosphatidylserine which is expressed on the surface of cells undergoing apoptosis. The Annexin V fluorescein isothiocyanate (FITC) kit was used according to the manufacturer's instructions (Oncogene Science). Cells were analysed 96 h post-irradiation and/or treatment with dexamethasone. Briefly, live cells were collected by trypsinization and stained with FITC-conjugated Annexin V and PI. Cells were then analysed by flow cytometry (Becton Dickinson FACStar Plus) to determine the percentage of Annexin-positive cells.

*DNA fragmentation*

Cells were treated as described for morphological analysis. At least $2 \times 10^6$ cells were lysed in 500 μl lysis buffer (5 mM Tris–HCl pH 7.4, 20 mM EDTA, 0.5% Triton X-100) at 4°C for 2 h. Cell debris was removed by centrifugation at 30 000 ***g*** for 30 min at 4°C. The supernatant was collected and treated with 0.5 mg $ml^{-1}$ RNAase at 50°C for 1 h, followed by 0.4 mg $ml^{-1}$ proteinase K in 1% sodium dodecyl sulphate (SDS) at 50°C for 1 h. The DNA was then extracted with choloroform/isoamyl alcohol (24:1) and precipitated overnight with 2.5 × the volume of 95% ethanol. The DNA was dissolved in 10 μl TE (10 mM Tris–HCl, pH 8.0, 1 mM EDTA) and subjected to gel electrophoresis on a 1% agarose gel in 1 × TAE buffer. Gels were stained with ethidium bromide, visualized by ultraviolet light and photographed using Polaroid 57 film.

### FACS analysis

Cells were treated as described above on day 0, collected at 12 h, 1 day, 2 days and 4 days following treatment and fixed in 100% cold methanol to permeablize the cells. Cells were washed in PBS/0.25% BSA/0.1% $NaN_3$ and incubated with either a mouse anti-human p53 primary antibody (Oncogene Science, Ab6) or an isotype-matched control antibody ($IgG_{2a}$); both were used at a final

 © 2000 Cancer Research Campaign concentration of 2 $\mu$g ml$^{-1}$ and cells were incubated for 30 min at room temperature. Ab6 binds to amino acids 37–45 on both mutant and wild-type p53 proteins. Cells were washed and incubated for 15 min at room temperature with a FITC-conjugated rabbit anti-mouse IgG at a final concentration of 300 $\mu$g ml$^{-1}$. Cells were analysed by flow cytometry on a Becton Dickinson FACStar Plus and the mean green fluorescence corresponding to p53 content was determined as the mean fluorescence of the test sample minus that of control immunoglobulin. The specificity of the p53 antibody (Oncogene Science, Ab6) was confirmed by Western blot analysis.

### Western blot

Protein was extracted from control cells, cells treated with 1 $\mu$M dexamethasone and cells irradiated at 6 Gy in the presence or absence of dexamethasone. Protein extraction was performed for 30 min on ice in RIPA buffer containing 250 mM NaCl, 50 mM Tris, 0.1% Nonldet P-40, 0.1% SDS, 0.5% sodium deoxycholate and the protease inhibitors leupeptin and phenyl methylsulphonyl fluoride (PMSF). Proteins were quantitated using the BioRad Detergent Compatible Protein Assay kit. Equal amounts of protein (15–20 $\mu$g) were diluted in Laemmli buffer containing $\beta$-mercaptoethanol and resolved on a 10% minigel by SDS-polyacrylamide gel electrophoresis (SDS-PAGE). Proteins were then transferred to a polyvinyl difluoride (PVDF) membrane (BioRad) using the NOVEX system followed by immunoblotting using an antibody to p53 (Oncogene Science Ab 6). The membrane was subsequently developed using enhanced chemiluminescence (ECL, Amersham).

### RESULTS

### Dexamethasone alters E6/E7 mRNA

C4-1, SW756 and C33-a cells were irradiated with 6 Gy in the presence or absence of 1 $\mu$M dexamethasone. Northern blot analysis revealed that transcription of the HPV E6/E7 was increased when C4-1 cells were treated with dexamethasone for 48 h while E6/E7 mRNA levels decreased in the SW756 cells (Figure 1A). C33a cells do not possess the HPV genome and were therefore negative for expression of HPV E6/E7 mRNA as determined by Northern blot (data not shown). The E6/E7 mRNA levels were normalized against the 18/28 S ribosomal RNA and the standard error of the mean densitometric units are shown (Figure 1B). It was determined that in comparison to control cells, C4-1 cells treated with dexamethasone alone or in combination with irradiation exhibited a five- to sevenfold induction of HPV E6/E7 mRNA. In contrast, the dexamethasone-treated and dexamethasone-treated plus irradiated SW756 cells showed a twofold reduction in HPV E6/E7 mRNA in comparison to untreated cells.

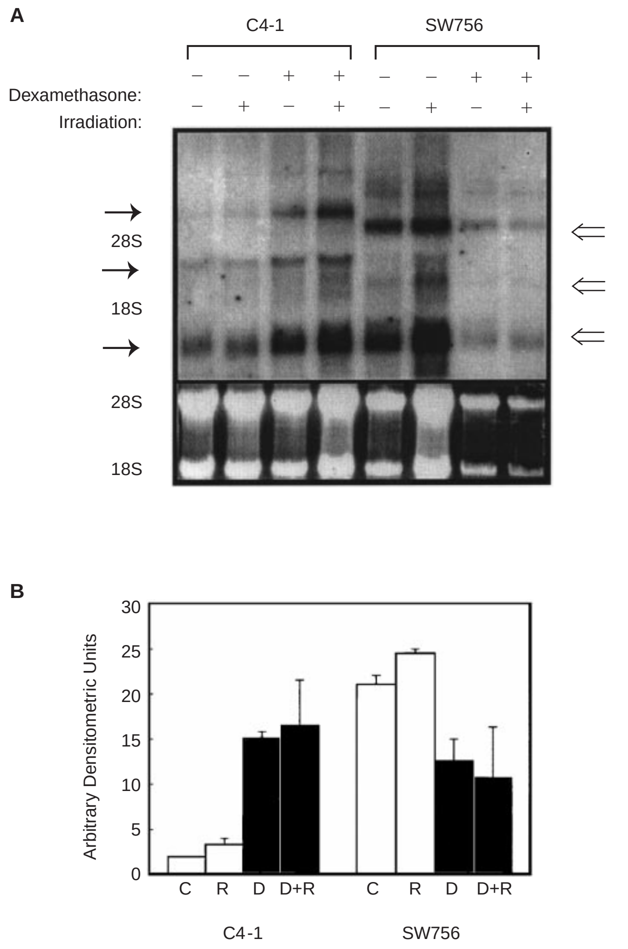

**Figure 1** Northern blot analysis of HPV 18 E6/E7 mRNA 48 h after treatment (**A**). In C4-1 cells, dexamethasone increases the E6/E7 mRNA level alone or in combination with irradiation by five- to sevenfold over that of control or irradiated cells as determined by densitometry. In SW 756 cells, expression of HPV E6/E7 mRNA was repressed twofold in the presence of dexamethasone alone or in combination with irradiation. Three separate experiments were performed and the standard error of the mean densitometric units is shown (**B**). Equal loading of RNA is demonstrated by the ribosomal RNA 18S and 28S bands. (HPV E6/E7 mRNA was normalized against the ribosomal RNA to determine the fold increase/decrease in expression.) The major HPV 18 transcripts are shown for C4-1 (→) and SW756 (⇐)

### Analysis of apoptosis

It has been shown that overexpression of E6 or E6/E7 results in abrogation of apoptosis (Pan and Griep, 1994; Thomas et al, 1996; Puthenveettil et al, 1997); however, these studies addressed the effect of E6/E7 on programmed cell death utilizing transgenic animals or cells that overexpress the viral genes through vector or adenoviral-mediated transfection. In keratinocytes infected with HPV, the viral genes E6/E7 are controlled by the same promoter; therefore, it is important to understand the effect of simultaneous manipulation of both E6 and E7. We hypothesized that up-regulation of E6/E7 in response to dexamethasone would diminish the potential for cervical cells to access apoptosis following $\gamma$ radiation. Therefore, the effect of glucocorticoids on radiation-induced apoptosis was determined as assessed by nuclear morphology and Annexin V staining (Figure 2). The control, untreated cells displayed homogeneously-stained DNA and a round, regularly shaped nucleus.

Irradiated cells displayed condensed or fragmented nuclei, both of which are hallmarks of apoptosis. The C4-1 cell line (in which dexamethasone increases the HPV E6/E7 genes) showed a time-dependent increase in the percent of apoptosis over a 4-day period (Figure 2A) and the level of radiation-induced apoptosis was decreased when dexamethasone was added to cell cultures prior to irradiation. These results were confirmed by Annexin V staining at

© 2000 Cancer Research Campaign

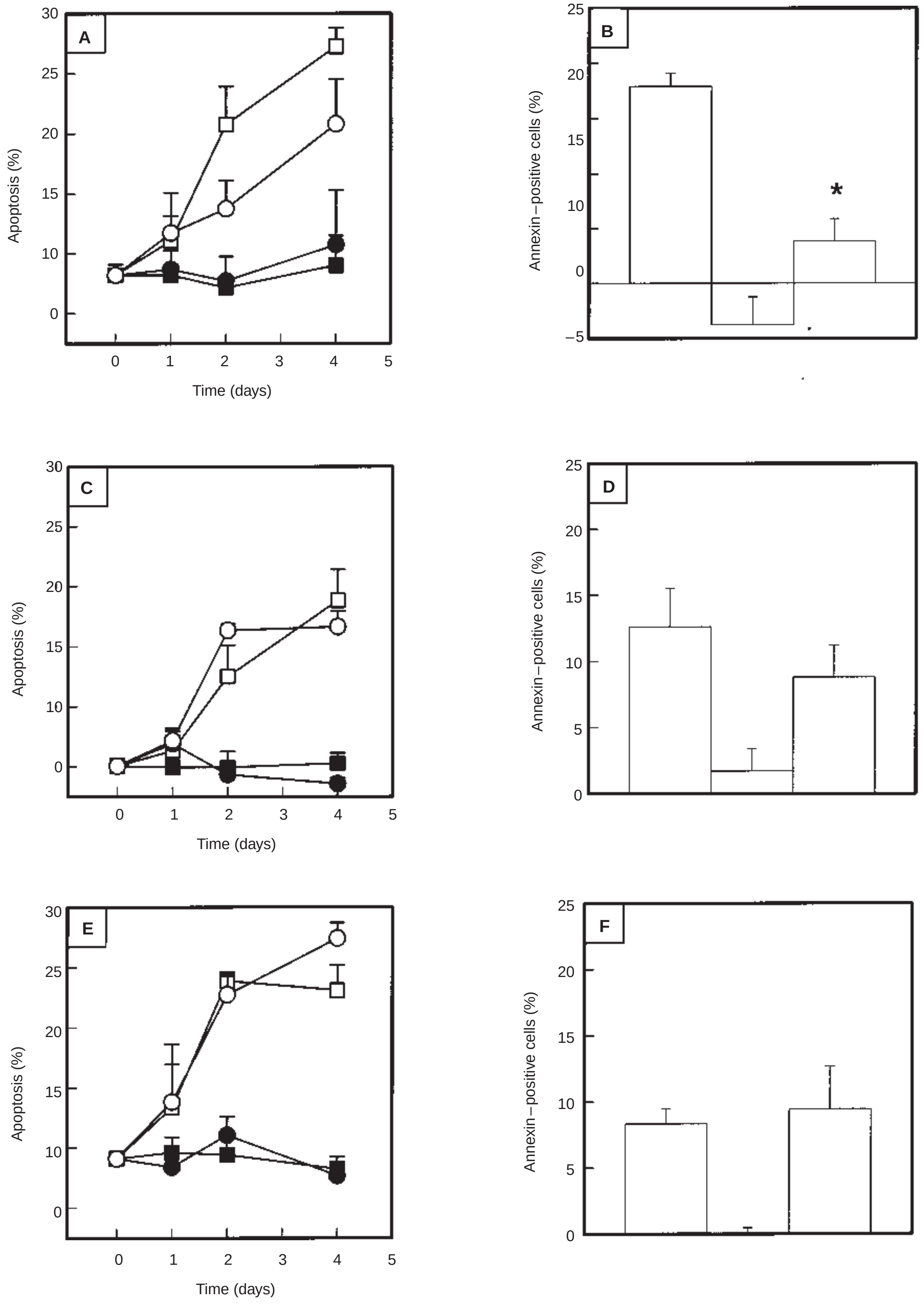

**Figure 2** Percent apoptosis in HPV-positive (**A, B** C4-1; **C, D** SW756) or HPV-negative (**E, F** C33a) cell lines as assessed by morphology over 4 days (**A, C, E**) and Annexin V staining at 4 days after treatment (**B, D, F**). The standard error of the mean of at least three separate experiments is shown. Radiation-induced apoptosis decreased in the C4-1 cells by the addition of 1 μM dexamethasone prior to irradiation. In C4-1 cells, Annexin V staining showed that at 4 days following treatment, the dexamethasone-mediated inhibition of apoptosis was significant (*$P < 0.01$, radiation versus radiation plus dexamethasone). In contrast, dexamethasone had no effect on the apoptosis of cells in which dexamethasone repressed HPV E6/E7 (SW 756) or cells which lack HPV. Cells are treated as follows: control (■); dexamethasone (●); radiation (□); radiation+dexamethasone (○)

 *© 2000 Cancer Research Campaign*

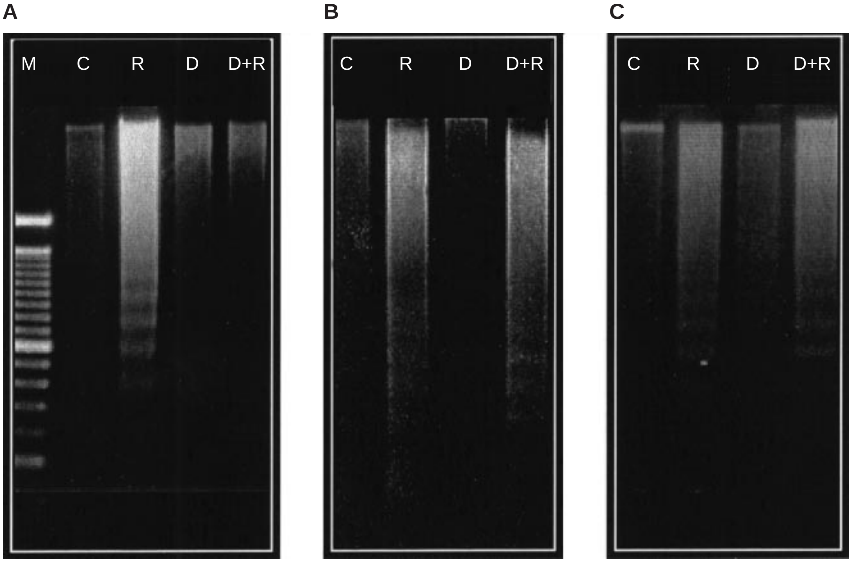

**Figure 3** Apoptosis was determined by DNA fragmentation analysis 4 days following treatment in C4-1 (**A**), SW756 (**B**) and C33-a (**C**) cells. Dexamethasone had no effect on radiation-induced apoptosis in cells that lack HPV (C33-a) or in cells in which HPV E6/E7 transcription is repressed following treatment with glucocorticoids (SW756). However, a marked inhibition of DNA fragmentation was observed in C4-1 cells irradiated in the presence of dexamethasone. Cells were untreated (C), treated with 6 Gy irradiation (R) or 1 μM dexamethasone (D) or both radiation and dexamethasone (D+R) as indicated; M: marker (100 bp ladder)

4 days post-treatment and the dexamethasone-mediated inhibition of apoptosis was found to be significant ($P < 0.01$) (Figure 2B). In contrast, dexamethasone did not alter the ability of the SW756 cells (in which dexamethasone represses the HPV E6/E7 genes) to undergo apoptosis following irradiation as cells irradiated alone or in the presence of dexamethasone displayed similar levels of apoptosis over a 4-day period (Figure 2 C, D). The C-33a cells which lack HPV also exhibited no change in the level of radiation-induced apoptosis when cells were pretreated with dexamethasone (Figure 2 E, F).

In order to assess a biochemical correlate of apoptosis in our system, the presence of internucleosomal cleavage was determined using the DNA 'laddering' assay. As a result of cell death by apoptosis, DNA is cleaved into multiples of 200 basepair fragments which correspond to cleavage of internucleosomal DNA. Analysis by gel electrophoresis revealed that DNA from apoptotic cells exhibited a characteristic ladder. By 4 days following irradiation, the C4-1 cells showed clear DNA laddering when cells were irradiated, but this effect was almost completely abrogated in cells that were treated with dexamethasone prior to irradiation. In contrast, dexamethasone had no effect on radiation-induced DNA fragmentation in either the SW756 or the C33-a cell lines (Figure 3 A–C).

### Effect of dexamethasone on p53 protein levels

Despite HPV infection of human cervical keratinocytes, p53 is capable of transactivating its target genes GADD45 and p21, which are involved in DNA repair processes or activation of the G1/S checkpoint (Butz et al, 1995, 1999). Therefore, within the HPV-containing cervical cell lines studied here, the p53 pathway is likely to be functional and may be regulated by radiation and/or dexamethasone treatment. In order to elucidate the molecular mechanism by which the C4-1 cells escape radiation-induced apoptosis in the presence of dexamethasone, we determined the effect of glucocorticoid treatment on the level of p53 protein expression in these cells (Figure 4A). As analysed by flow cytometry, C4-1 cells treated with 1 μM dexamethasone alone exhibited decreased expression of p53 protein for up to 4 days in comparison to control cells. While irradiated cells showed an approximately

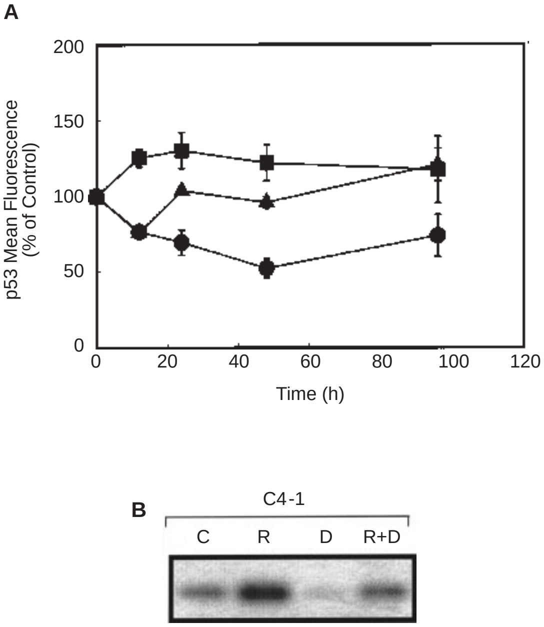

**Figure 4** Levels of p53 protein were determined in C4-1 cells by flow cytometry (**A**) following treatment with dexamethasone (●), radiation (■) or both dexamethasone and irradiation (▲) (The error bars are the standard error of the mean of four separate experiments.) Protein levels are expressed as a percent of control. By 24 h following treatment, dexamethasone decreased while radiation increased p53 by approximately 40% respectively. p53 levels remained near control values followed by dexamethasone and radiation treatment. These data were confirmed at 48 h following treatment by Western blot analysis (**B**)

*© 2000 Cancer Research Campaign*

50% increase in p53 expression by 48 h after irradiation, cells irradiated in the presence of dexamethasone exhibited p53 expression similar to that of control cells, an effect intermediate between that observed with either dexamethasone or irradiation alone. To confirm the specificity of the flow cytometry data, p53 protein was also analysed by Western blot at 24 h following treatment which revealed the same trend in p53 expression (Figure 4B). C-33a cells possess mutant p53 protein which is highly overexpressed as determined by flow cytometry and Western blot analysis (data not shown) while SW756 cells cultured in dexamethasone exhibited decreased HPV E6/E7 mRNA and increased p53 levels as determined by Western blot (Von Knebel Doeberitz et al, 1994). Therefore, it appears that loss of p53 expression due to dexamethasone-mediated up-regulation of HPV E6/E7 is associated with decreased apoptosis following γ irradiation.

### Analysis of p21 mRNA expression

The data presented indicate that exposure to dexamethasone modifies both upstream (p53) and downstream (fragmentation) components of the apoptotic pathway in C4-1 cells. In order to determine whether p53 is functional, p21, a cyclin-dependent kinase inhibitor which is transcriptionally regulated by p53, was examined (el-Deiry et al, 1993; Harper et al, 1993). It was found that p21 mRNA is regulated in the same manner as p53 (Figure 5), which is consistent with the proposal that steroid-mediated changes in the apoptotic pathway involve p53 and its downstream targets.

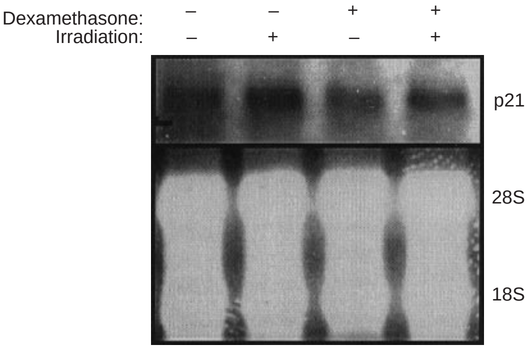

**Figure 5** Northern blot analysis of p21 mRNA was performed following treatment with radiation, dexamethasone or both at 48 h following treatment. In comparison to untreated cells, p21 mRNA increased following irradiation while dexamethasone treatment led to decreased p21 mRNA. Cells that were irradiated in the presence of dexamethasone exhibited decreased p21 in comparison to irradiated cells. p21 mRNA was normalized against the 28/18S ribosomal RNA

### DISCUSSION

Our data provide support for the notion that in HPV-transformed cells, access to apoptosis appears dependent upon p53 function. The p53 protein facilitates apoptotic cell death in response to DNA damaging agents such as radiation or chemotherapeutic drugs (Yonish-Rouach et al, 1991; Shaw et al, 1992). Since p53 is an important regulator of apoptosis, changes in p53 status in HPV-infected cells may alter access to programmed cell death. Here, dexamethasone treatment prior to irradiation resulted in a significant decrease in the level of radiation-induced apoptosis and p53 protein in C4-1 cells, concomitant with an increase in HPV E6/E7 mRNA. For this reason, dexamethasone is proposed to contribute to a decrease in apoptosis through E6-mediated degradation of p53 in C4-1 cells. In contrast, neither the HPV-negative C-33a cells, nor the SW756 cells that repress HPV E6/E7 and induce p53 in the presence of dexamethasone, exhibited diminished apoptosis following irradiation and steroid treatment (Von Knebel Doeberitz et al, 1994).

Various studies demonstrating that HPV E6 and E7 can alter access to apoptosis support our findings that dexamethasone-mediated up-regulation of E6/E7 results in inhibition of apoptosis. While both E6 and E6/E7 have been shown to abrogate p53 dependent cell cycle arrest (Kessis et al, 1993; Thomas et al, 1996; Wang et al, 1996; Hickman et al, 1997), the viral proteins differ in their abilities to promote or inhibit apoptosis. In transgenic mice, E7 induced lens cell proliferation and apoptosis dependent upon its association with Rb and Rb-like proteins while apoptosis-like DNA degradation was reduced in E6 × E7 or inhibited in E6 transgenic mice (Pan and Griep, 1994, 1995). Also, p53-dependent and -independent apoptosis have been demonstrated in Rb knockout mice (Morgenbesser et al, 1994) which suggests that loss of Rb is the primary function of E7 that enhances apoptosis. E7 has been shown to prime or sensitize cells to apoptosis in response to an appropriate stimulus (Puthenveettil et al, 1996; Iglesias et al, 1998; Stoppler et al, 1998). While E7-transduced keratinocytes exhibited increased apoptosis and p53 levels, cells that express E6 or E6/E7 fail to undergo apoptosis following DNA damage suggesting that E6 has compromised p53 function by ubiquitin-dependent degradation (Puthenveettil et al, 1996; Thomas et al, 1996). Taken together, these data support the present study by showing that through inactivation of p53, up-regulation of E6 or E6/E7 can abrogate apoptosis during development or following DNA damage.

Mutation of p53 can influence the ability of radiation to induce apoptosis, thus p53 appeared to be necessary for radiation-induced programmed cell death (Clarke et al, 1993; Lowe et al, 1993). However, it has been shown that apoptosis occurs in cells with wild-type p53 and in cells which possess mutations in, or lack p53 following irradiation (Radford, 1994; Strasser et al, 1994; Bracey et al, 1995). Despite HPV infection of human cervical keratinocytes, p53 is capable of transactivation of its downstream target genes GADD45 and p21 as shown by others (Butz et al, 1995, 1999) and p21, as shown here. Therefore, in HPV-infected cervical epithelial cells, the presence of the E6 protein may not completely abrogate the apoptotic pathway through loss of p53 as cells still activate p53-dependent genes. These studies support the presence of a functional p53 in our system as we have demonstrated that regulation of p53 and p21 correspond to observed changes in apoptotic cell death.

Glucocorticoids have been shown to readily cause apoptosis in cells of the lymphoid system. Thymocytes from p53 knockout mice still access apoptosis in response to dexamethasone, thus, apoptosis in response to glucocorticoids occurs independent of p53 (Clarke et al, 1993; Lowe et al, 1993). However, through mechanisms which remain unclear, dexamethasone has also been shown to protect cells from apoptosis in various systems. Dexamethasone affords cytoprotection to malignant but not untransformed glial cells, which has been correlated with a p53-independent increase in p21 protein (Naumann et al, 1998). In contrast, dexamethasone protected mouse embryonic fibroblasts from drug-mediated cytotoxicity but only in cells which were deficient in p21. Thus, the mechanism by which dexamethasone

 © 2000 Cancer Research Campaign protects cells from apoptosis appears to be cell type-specific and may or may not involve p53 and its downstream targets. The data presented here support a clear mechanism for dexamethasone-dependent inhibition of apoptosis involving modulation of HPV E6 and p53 in cervical carcinoma cells.

Steroid-mediated inhibition of apoptosis in human cervical carcinoma cells may have clinical impact. Like dexamethasone, progesterone has been shown to activate HPV gene expression by binding to the same GRE. Progesterone-induced elevation in viral RNA was blocked by the progestin antagonist RU486 (Mittal et al, 1993). Therefore, endogenous levels of steroids and those existing as a consequence of taking oral contraceptives may induce HPV gene transcription and contribute to tumour progression (Stern et al, 1977; Zur Hausen, 1991). Since endogenous or administered hormones can activate GRE-driven increase in HPV transcription, HPV-positive tumours may also be radioresistant and less susceptible to apoptosis following irradiation. If true, the administration of steroid/receptor antagonists, such as RU486, might be beneficial prior to radiotherapy to enhance tumour cell death through the apoptotic pathway.

### REFERENCES

Almasan A, Linke SP, Paulson TG, Huang L and Wahl GM (1995) Genetic instability as a consequence of inappropriate entry into and progression through S phase. *Cancer Metast Rev* **14**: 59–73

Band V, DeCaprio JA, Delmolino L, Kulesa V and Sager R (1991) Loss of p53 protein in human papillomavirus type 16 E6-immortalized human mammary epithelial cells. *J Virol* **65**: 6671–6676

Bracey TS, Miller JC, Preece A and Paraskeva C (1995) Gamma-radiation-induced apoptosis in human colorectal adenoma and carcinoma cell lines can occur in the absence of wild type p53. *Oncogene* **10**: 2391–2396

Butz K, Shahabeddin, L, Geisen C, Spitovsky P, Ullmann A and Hoppe-Seyler F (1995) Functional p53 protein in human papillomavirus-positive cancer cells. *Oncogene* **10**: 927–936

Butz K, Geisen C, Ullmann A, Spitovsky D and Hoppe-Seyler F (1996) Cellular responses of HPV-positive cancer cells to genotoxic anti-cancer agents, repression of E6/E7-oncogene expression and induction of apoptosis. *Int J Cancer* **68**: 506–513

Butz K, Whitaker N, Denk C, Ullmann A, Geisen C and Hoppe-Seyler F (1999) Induction of the p53-target gene GADD45 in HPV-positive cancer cells. *Oncogene* **18**: 2381–2386

Clark AR, Purdie CA, Harrison DJ, Morris RG, Bird CC, Hooper ML and Wyllie AH (1993) Thymocyte dependent apoptosis induced by p53-dependent and independent pathways. *Nature* **362**: 849–852

Crook T, Tidy JA and Vousden KH (1991) Degradation of p53 can be targeted by HPV E6 sequences distinct from those required for p53 binding and trans-activation. *Cell* **67**: 547–556

Demers GW, Foster SA, Halbert CL and Galloway DA (1994) Elevated wild-type p53 protein levels in human epithelial cell lines immortalized by the human papillomavirus type 16 E7 gene. *Proc Natl Acad Sci USA* **91**: 4382–4386

Dyson N, Howley PM, Munger K and Harlow E (1989) The human papilloma virus-16 E7 oncoprotein is able to bind to the retinoblastoma gene product. *Science* **243**: 934–936

el-Deiry WS, Tokino T, Velculescu VE, Levy DB, Parsons R, Trent JM, Lin D, Mercer WE, Kinzler KW and Vogelstein B (1993) WAF1, a potential mediator of p53 tumor suppression. *Cell* **75**: 817–825

Gloss B, Bernard HU, Seedorf K and Klock G (1991) The upstream regulatory region of the human papillomavirus contains an E2 protein independent enhancer which is specific for cervical carcinoma and regulated by glucocorticoid hormones. *EMBO* **6**: 3735–3743

Harper JW, Adami GR, Wei N, Keyomarsi K and Elledge SJ (1993) The p21 Cdk-interacting protein Cip1 is a potent inhibitor of G1 cyclin-dependent kinases. *Cell* **75**: 805–816

Hickman ES, Bates S and Vousden KH (1997) Perturbation of the p53 response by human papillomavirus type 16 E7. *J Virol* **71**: 3710–3718

Hoppe-Seyler F and Butz K (1994) Cellular control of human papillomavirus oncogene transcription. *Mol Carcinogenesis* **10**: 134–141

Iglesias M, Yen K, Gaiotti D, Hildesheim A, Stoler MH and Woodworth CD (1998) Human papillomavirus type 16 E7 protein sensitizes cervical keratinocytes to apoptosis and release of interleukin-1alpha. *Oncogene* **17**: 1195–1205

Kangas A, Nicholson DW and Hottla E (1998) Involvement of CPP32/Caspase-3 in c-Myc-induced apoptosis. *Oncogene* **16**: 387–398

Kessis TD, Slebos RJ, Nelson WG, Kastan MB, Plunkett SM, Han AT, Lorincz L, Hedrick L and Cho KL (1993) Human papillomavirus 16 E6 expression disrupts the p53-mediated cellular response to DNA damage. *Proc Natl Acad Sci USA* **90**: 3988–3992

Lazo PA (1999) The molecular genetics of cervical carcinoma. *Br J Cancer* **80**: 2008–2018

Lechner MS and Laimins LA (1994) Inhibition of p53 DNA binding by human papillomavirus E6 proteins. *J Virol* **68**: 4262–4273

Lowe SW and Ruley HE (1993) Stabilization of the p53 tumor suppressor is induced by adenovirus E1A and accompanies apoptosis. *Genes Dev* **7**: 535–545

Lowe SW, Schmitt EA, Smith SW, Osbourne BA and Jacks T (1993) p53 is required for radiation-induced apoptosis in mouse thymocytes. *Nature* **362**: 847–849

Mittal R, Tsutsumi K, Pater A and Pater MM (1993) Human papilloma virus type 16 expression in cervical keratinocytes: role of progesterone and glucocorticoid hormones. *Obs Gyn* **81**: 5–12

Morgenbesser SD, Williams BO, Jacks T and DePinho RA (1994) *Nature* **371**: 72–74

Naumann U, Durka S and Weller M (1998) Dexamethasone-mediated protection from drug cytotoxicity, association with p21 WAF1/CIP1 protein accumulation? *Oncogene* **7**: 1567–1575

Pan H and Griep AE (1994) Altered cell cycle regulation in the lens of HPV-16 E6 or E7 transgenic mice: implications for tumour suppressor gene function in development. *Genes Dev* **7**: 1285–1299

Pan H and Griep AE (1995) Temporally distinct patterns of p53-dependent and independent apoptosis during mouse lens development. *Genes Dev* **9**: 2157–2169

Puthenveettil JA, Frederickson SM and Reznikoff CA (1996) Apoptosis in human papillomavirus 16 E7-, but not E6-immortalised human uro-epithelial cells. *Oncogene* **13**: 1123–1131

Radford IR (1994) p53 status, DNA double-strand break repair proficiency, and radiation response of mouse lymphoid and myeloid cell lines. *Int J Radiat Biol* **66**: 557–560

Scheffner M, Werness BA, Huibregtse JM, Levine AJ and Howley PM (1990) The E6 oncoprotein encoded by HPV types 16 and 18 promotes the degradation of p53. *Cell* **63**: 1129–1136

Scheffner M, Munger K, Byrne JC and Howley PM (1991) The state of the p53 and retinoblastoma genes in human cervical carcinoma cell lines. *Proc Natl Acad Sci USA* **88**: 5523–5527

Shaw P, Bovey R, Tardy S, Sahli R, Sordat B and Costa J (1992) Induction of apoptosis by wildtype p53 in a human colon tumor-derived cell line. *Proc Natl Acad Sci USA* **89**: 4495–4499

Stern E, Forsythe AB, Youkeles L and Coffelt CF (1977) Steroid contraceptive use and cervical dysplasia: increased risk of progression. *Science* **196**: 1460–1462

Stoppler H, Stoppler MC, Johnson E, Simbulan-Rosenthal CM, Smulson ME, Iyer S, Rosenthal DS and Schlegel R (1998) The E7 protein of human papillomavirus type 16 sensitizes primary human keratinocytes to apoptosis. *Oncogene* **17**: 1207–1214

Strasser A, Harris AW, Jacks T and Cory S (1994) DNA damage can induce apoptosis in proliferating lymphoid cells via p53-independent mechanisms inhibitable by Bcl-2. *Cell* **79**: 329–339

Thomas M, Nassimi P, Jenkins J and Banks L (1995) HPV-18 E6 mediated inhibition of p53 DNA binding activity is independent of E6 induced degradation. *Oncogene* **10**: 261–268

Thomas M, Matlashewski G, Pim D and Banks L (1996) Induction of apoptosis by p53 is independent of its oligomeric state and can be abolished by HPV-18 E6 through ubiquitin mediated degradation. *Oncogene* **10**: 109–115

Von Knebel Doeberitz M, Tilman O, Schwarz E and Gissmann L (1988) Correlation of modified human papilloma virus early gene expression with altered growth properties in C4-1 cervical carcinoma cell lines. *Cancer Res* **48**: 3780–3786

Von Knebel Doeberitz M, Koch S, Drzonek H and ZurHausen H (1990) Glucocorticoid hormones reduce the expression of major histocompatibility class I antigens on human epithelial cells. *Eur J Immunol* **20**: 35–40

Von Knebel Doeberitz M, Bauknecht T, Bartsch D, ZurHausen H (1991) Influence of chromosomal integration on glucocorticoid-regulated transcription of growth-stimulating papillomavirus genes E6/E7 in cervical carcinoma cells. *Proc Natl Acad Sci USA* **88**: 1411–1415

Von Knebel Doeberitz M, Ritmuller C, Zur Hausen H and Durst M (1992) Inhibition of tumorigenicity of cervical cells in nude mice by HPV E6/E7 anti-sense RNA. *Int J Cancer* **55**: 831–834

*© 2000 Cancer Research Campaign*

Von Knebel Doeberitz M, Ritmuller C, Aengeneyndt F, Jansen-Durr P and Spitovsky D (1994) Reversible repression of papillomavirus oncogene expression in cervical carcinoma cells: consequences for the phenotype and E6-p53 and E7-pRb interactions. *J Virol* **68**: 2811–2821

Walboomers JM, Jacobs MV, Manos MM, Bosch FX, Kummer JA, Shah KV, Snijders PJ, Peto J, Meijer CJ and Munoz N (1999) Human papillomavirus is a necessary cause of invasive cervical cancer worldwide. *J Pathol* **189**: 12–19

Wang Y, Okan I, Pokrovskaja K and Wiman KG (1996) Abrogation of p53-induced G1 arrest by the HPV16 E7 protein does not inhibit p53-induced apoptosis. *Oncogene* **12**: 2731–2735

Werness BA, Levine AJ and Howley PM (1990) Association of HPV types 16 and 18 E6 proteins with p53. *Science* **248**: 76–79

White E (1996) Life, death, and the pursuit of apoptosis. *Genes and Dev* **10**: 1–15

Yonish-Rouach E, Resnitzky D, Lotem J, Sachs L, Kimchi A and Oren M (1991) Wildtype p53 induces apoptosis of myeloid leukaemic cells that is inhibited by interleukin-6. *Nature* **352**: 345–347

Yu Y and Little JB (1998) p53 is involved in but not required for ionizing radiation-induced caspase-3 activation and apoptosis in human lymphoblast cell lines. *Cancer Res* **58**: 4277–4281

Zur Hausen H (1991) Human papillomaviruses in the pathogenesis of anogenital cancer. *Virology* **184**: 9–13

 *© 2000 Cancer Research Campaign*
